# Comparison of Causes of Mortality Between Hospitalized Unsheltered Homeless Patients and Non-Homeless Sex and Age-Matched Controls: A Retrospective Case-Control Study

**DOI:** 10.3389/ijph.2024.1607642

**Published:** 2024-09-17

**Authors:** Juraj Smaha, Jakub Falat, Andrea Gažová, Martin Kužma, Ján Kyselovič, Michal Palkovič, Roman Kuruc, Pavel Babál, Juraj Payer, Peter Jackuliak

**Affiliations:** ^1^ 5th Department of Internal Medicine, Faculty of Medicine, Comenius University, Bratislava, Slovakia; ^2^ Institute of Pharmacology and Clinical Pharmacology, Faculty of Medicine, Comenius University, Bratislava, Slovakia; ^3^ Institute of Pathological Anatomy, Faculty of Medicine, Comenius University, Bratislava, Slovakia; ^4^ The Health Care Surveillance Authority, Bratislava, Slovakia; ^5^ Institute of Forensic Medicine, Faculty of Medicine, Comenius University, Bratislava, Slovakia

**Keywords:** homelessness, roofless, rough sleeping, mortality, infection

## Abstract

**Objectives:**

Roofless individuals represent the most severe category of homelessness. Their clinical characteristics and mortality patterns in Central and Eastern Europe are little known.

**Methods:**

A single-center retrospective case-control study at the internal medicine department in Bratislava, Slovakia was conducted. 5694 mortality records from 2010 to 2023 were screened, and 141 (118 men, 23 women) roofless individuals were identified. Patients were sex- and age-matched, with 141 patients from the cohort of non-homeless deceased patients.

**Results:**

Compared to controls, roofless people had a higher incidence of immobility (p = 0.02) and hypothermia (p < 0.0001) at admission. 83% of the roofless people were men, and 59% of the roofless people died before reaching old age (60+). Homeless men died more often from infectious disease (p = 0.02), pneumonia being the most common one (60%). Men from the control group died more often from liver diseases (p = 0.03). There were no significant differences in the causes of mortality between women.

**Conclusion:**

These findings could help to reduce the invisibility of the issue of massive premature mortality amongst homeless populations and roofless individuals, in particular.

## Introduction

Homeless people are a vulnerable group that accounts for approximately 1% of the population; however, with increasing incidence [1]. It is estimated that at least 700,000 homeless people sleep rough or in temporary emergency accommodation in the European Union on a given night; however, the exact numbers of homeless people are largely unknown [2]. Specifically, in Slovakia, our national legislature does not define “homelessness” and “homeless people” [3]. According to the 2011 Population and Housing Census, there were 23,483 homeless persons in Slovakia (0.4% of the total population). Alarmingly, according to the last census, the number of homeless people increased between 2011 and 2021 almost threefold, to 71,076 people [4]. At the same time, Slovakia’s overall population remained stable between 2011 and 2021 (5,398,109 people in 2011 vs. 5,442,759 people in 2021) [5]. However, this number of homeless people refers only to persons living in long-term transitional shelters. As a result, the core categories of the homeless population – people living rough or people staying in night shelters – were not included [6]. In Bratislava, the capital city, it is estimated that approx. 3,000 people are homeless on any given night [7].

A systematic review of the literature about critical illness in homeless persons published in 2014 laid out several important topics in this area of research [8]. Homelessness is the most extreme form of social exclusion, and is associated with a high burden of disease. In comparison to non-homeless people, homeless people have increased substance abuse and psychiatric, chronic, and infectious disorders [8]. Homeless people present more frequently to hospitals with advanced courses of the disease [8]. They also have, in general, worse outcomes and access to medical care [9, 10]. Homeless people experience excess mortality [11], and homelessness has been identified as an independent risk factor for mortality [12].

The issue of homelessness is often deprioritized as a focus for research. Therefore, some authors emphasize the need for a focused study in the area of acutely ill homeless patients [8]. Similarly, the European Commission stressed the essential need for strengthening evidence on homelessness to tackle the problem [13].

The definition of homelessness differs according to various documents, laws, and geographical regions. The European Federation of Organizations Working with the People who are Homeless (FEANTSA) recognizes four main concepts of homelessness: Rooflessness, Houselessness, Insecure Housing, and Inadequate Housing [14]. Rooflessness represents the most severe category, comprising people living in the streets or public spaces without a shelter, which can be defined as living quarters, and people with no usual place of residence who use overnight shelter [14].

To this date, studies concerning the causes of mortality of homeless persons have been carried out predominantly in large American cities and Western Europe. This problem is much less studied in Central and Eastern Europe, and there is no such study in Slovakia. This study aimed to describe causes of mortality among a cohort of homeless adults hospitalized for an acute illness in the internal medicine department of a University Hospital in Bratislava, Slovakia, compared to those of age- and sex-matched non-homeless patients.

## Methods

This was a single-center retrospective case-control study at the 5th Department of Internal Medicine, Comenius University and University Hospital Bratislava, Slovakia.

**FIGURE 1:**
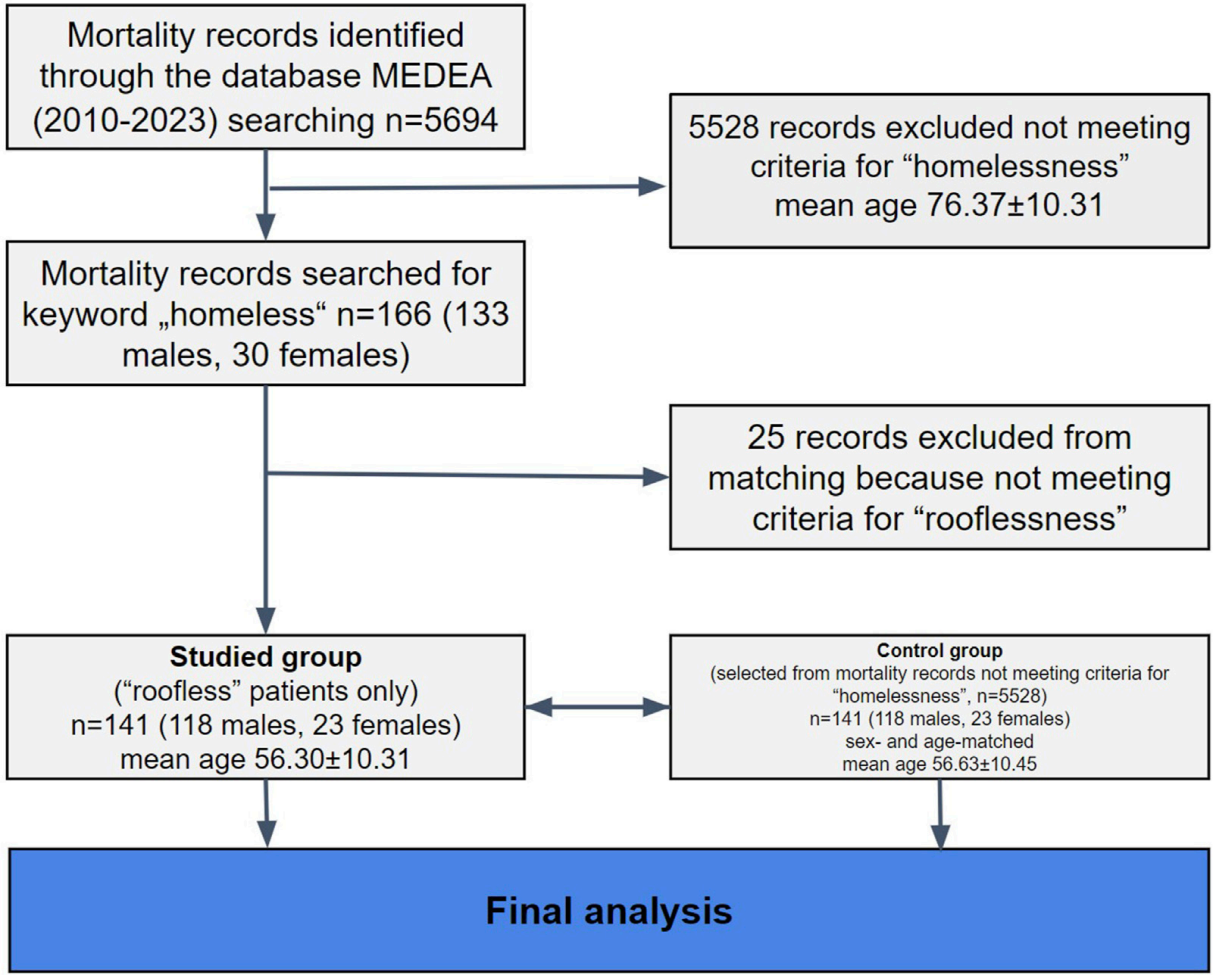
Study flowchart (Bratislava, Slovakia. 2024).

Hospital files of all deceased patients hospitalized for an acute illness in the internal medicine department were reviewed. Mortality data were obtained from the hospital information system MEDEA database between January 1, 2010, and December 31, 2023 (n = 5694). Mortality records were first screened for the keyword “casus socialis” (n = 166). “Casus socials” is a term not commonly used in English literature, but in Slovakia, it often refers to a patient who is a homeless rough sleeper, i.e., a person with no usual place of residence who uses overnight shelter. However, this term is often also used for patients with poor hygiene status, abuse of substances, or without a regular job (or combinations of these factors). Therefore, after closer reading, 25 records were excluded, not meeting the criteria for homeless rough sleepers. Finally, only patients meeting FEANTSA criteria for rooflessness - people living in the streets or public spaces without a shelter that can be defined as living quarters and people with no usual place of residence who use overnight shelter [14], were analyzed. Homeless people meeting the criteria for rooflessness are in the literature often described as “rough sleepers,” “people sleeping rough,” or “unsheltered people,” and these terms can be used interchangeably. A total of 141 patients (118 males, 23 females) meeting these criteria for rooflessness were identified. These patients were then sex- and age-matched with 141 patients (118 males, 23 females) from the cohort of hospitalized non-homeless deceased patients. Patients were first matched for sex, then for age ±1 year. If several options were available for the match, patients with the closest length of hospitalization were chosen. The study flowchart is displayed in Figure 1.

Demographic characteristics, comorbidities, laboratory markers, and causes of death were collected from electronic medical records and discharge summaries by two physicians using a standardized approach. Causes of death were identified from hospital death records using the International Classification of Diseases 11th Edition. The most prevalent causes of death were divided into six main clusters: “Infectious,” “Liver,” “Cardiovascular,” “Massive gastrointestinal bleeding,” “Oncology,” and “Other.” The most prevalent clusters were further analyzed, and specific diseases were compared between homeless and non-homeless patients.

Two scoring systems, Child-Pugh and the MELD Na score (Model for End-Stage Liver Disease), were used to stratify and compare patients with cirrhosis [15].

Autopsy records were obtained from The Healthcare Surveillance Authority. They included the following information: major diagnoses and cause of death as described in the autopsy records, age, sex, length of hospital stay, and clinical diagnoses. Diagnostic discrepancies were independently evaluated by two physicians and were classified according to Goldman et al. [16]. There are five classes of criteria for discrepancies between clinical and pathological diagnosis. Class V means absolute agreement, classes IV and III are considered minor, and classes II and I are deemed major discrepancies.

The study was conducted in accordance with the Declaration of Helsinki and approved by the Institutional Ethics Committee of University Hospital Bratislava (ethical approval code: EK0112024).

All statistical analyses were performed using GraphPad Prism version 10.2.1 (GraphPad Software, San Diego, California, United States). The baseline characteristics of the homeless and non-homeless patients were compared. Continuous variables were presented as the mean ± standard deviation (SD). Categorical variables were presented as numbers with percentages, and Fisher’s exact test was used to analyze the significance of the differences. The unpaired Student t-test with Welch’s correlation and Mann-Whitney test was used to analyze the significance of the differences between homeless and non-homeless people. P values <0.05 were considered statistically significant.

## Results

A total of 141 sex- and age-matched homeless persons were analyzed. The mean age of the homeless cohort was 56 ± 10.31 years compared to 76.37 ± 12.20 years in the hospitalized non-homeless cohort (see Figure 1). 83% of the homeless patients were men. 6% of homeless persons died in early adulthood (20–39 years) and 52% in middle adulthood (40–59 years). Homeless rough sleepers represent 2.5% of all deaths in the internal medicine department during the analyzed period (13 years). Baseline demographic and clinical characteristics between the homeless and sex- and age-matched non-homeless persons are displayed in Table 1.

**TABLE 1:** Baseline characteristics and differences between homeless and non-homeless patients (Bratislava, Slovakia. 2024).

Variable	Homeless (n = 141)	Non-homeless (n = 141)	P-value
Demographics			
Males/females, n (%)	118/23 (84/16)	118/23 (84/16)	N/A
Age of death (years)	56.30 ± 10.31	56.63 ± 10.45	0.39
Duration of hospitalization (days)	5 ± 6	6 ± 7	0.03
Number of comorbidities, n	2 ± 2	3 ± 2	<0.0001
Chronic heart failure, n (%)	27 (19)	50 (35)	0.003
Coronary artery disease, n (%)	23 (16)	53 (38)	<0.0001
Arterial hypertension, n (%)	35 (25)	72 (51)	<0.0001
Diabetes mellitus, n (%)	13 (9)	43 (30)	<0.0001
Atrial fibrillation, n (%)	18 (13)	28 (20)	0.15
Chronic kidney disease, n (%)	25 (18)	61 (43)	<0.0001
Chronic pulmonary disease, n (%)	15 (11)	26 (18)	0.09
Malignancy, n (%)	14 (10)	28 (20)	0.03
Chronic liver disease, n (%)	58 (41)	55 (39)	0.8
Risk factors			
Alcohol abuse, n (%)	119 (84)	69 (49)	<0.0001
Smoker, n (%)	116 (82)	80 (57)	<0.0001
Intravenous drug user, n (%)	15 (11)	8 (6)	0.19
Selected clinical and laboratory characteristics on admission			
Impaired consciousness, n (%)	67 (48)	62 (44)	0.63
Impaired mobility, n (%)	90 (64)	69 (49)	0.02
Hypothermia, n (%)	23 (16)	0 (0)	<0.0001
Warm months (april-september)/Cold months (october-march) admissions	54(38)/87(62)	59(42)/82(58)	0.63
Hepatitis B infection, n (%)	4 (3)	5 (4)	>0.9
Hepatitis C infection, n (%)	21 (15)	10 (7)	0.055
Humane imunodeficiency virus, n (%)	1 (1)	0 (0)	>0.9
Syphyllis, n (%)	4 (3)	2 (1)	0.68
Ectoparasites, n (%)	14 (10)	0 (0)	<0.0001
Level of care			
Higher level of oxygen delivery (high-flow oxygen or mechanical ventilation), n (%)	15 (11)	24 (17)	0.17
Vasopressors, n (%)	70 (49)	83 (59)	0.15
Intravenous antibiotics, n (%)	116 (82)	125 (87)	0.18
Hemotherapy, n (%)	31 (22)	39 (28)	0.33
Ultrasound (abdomen, limb), n (%)	81 (57)	82 (58)	>0.9
Echocardiography, n (%)	17 (12)	21 (15)	0.60
Computed tomography (chest, abdomen, head), n (%)	64 (45)	43 (30)	0.01
Gastrointestinal endoscopy, n (%)	16 (11)	21 (15)	0.48
Diagnostic/therapeutic puncture of fluid (peritoneal, pleural), n (%)	16 (11)	25 (18)	0.17

**FIGURE 2:**
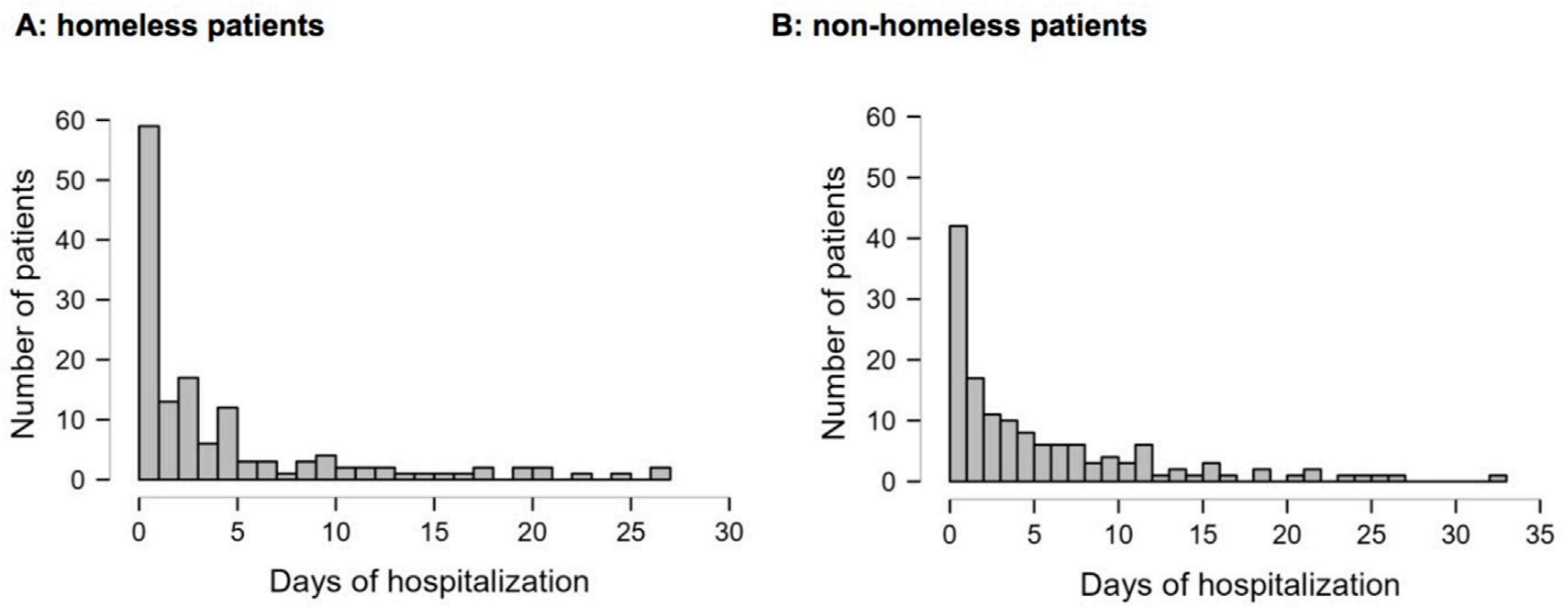
Length of hospital stay before death of homeless **(A)** and matched cohort of non-homeless **(B)** patients (Bratislava, Slovakia. 2024).

The mean duration of hospitalization in the homeless patients group was significantly shorter than in the non-homeless group (5 ± 6 vs. 6 ± 7, p = 0.03). Most patients died in the first 5 days of hospitalization (see Figure 2).

Non-homeless patients had significantly higher numbers of comorbidities. Of them, coronary artery disease, diabetes mellitus, arterial hypertension, and chronic kidney disease were highly significantly more prevalent in the non-homeless group of patients (all p < 0.0001). The most prevalent comorbidity in the group of homeless patients was chronic liver disease (41%), followed by arterial hypertension (25%) and chronic heart failure (19%). In the non-homeless group, the most prevalent comorbidity was arterial hypertension (51%), followed by chronic kidney disease (43%) and chronic liver disease (39%).

Smoking and alcohol abuse were highly significantly more prevalent in the homeless group (p < 0.0001). Intravenous drug users were not more prevalent in the homeless group. Both groups had no significant difference in chronic liver disease or chronic pulmonary disease.

Regarding clinical status on admission, homeless patients were more likely to be with impaired mobility (p = 0.02) and ectoparasites (p < 0.0001). There was no difference in hepatitis B (HBV), human immunodeficiency virus (HIV), and syphilis status between both groups. Hepatitis C (HCV) positive patients were more prevalent in the homeless group, which was borderline statistically significant (p = 0.055). Most patients from both groups (62% vs. 58%, p = 0.62) were admitted during cold months (October-March). Hypothermia on admission occurred more frequently in homeless patients in comparison to non-homeless matched cohorts (p < 0.0001).

There was no difference in the level of care between homeless and non-homeless patients regarding vasopressors, intravenous antibiotics, hemotherapy, diagnostic puncture and standard imaging except for computed tomography, which was more frequently requested for homeless patients (p = 0.01).

The most common category of death among homeless patients were infectious causes (48%), liver causes (16%), and cardiovascular causes (13%) (Table 2). Homeless men most commonly died because of infectious (49%), cardiovascular (15%), and liver (14%) problems. Homeless women died because of infectious (43%), liver (30%), and other issues (17%) (notably, the sample size of homeless women was small; each death accounts for a 4% change given the small denominator).

**TABLE 2:** Differences in main mortality clusters between homeless and non-homeless patients in both sexes, males and females, respectively (Bratislava, Slovakia. 2024).

	All patients (n = 282)			Males (n = 236)			Females (n = 46)		
Cause of death	Homeless (n = 141)	Non-homeless (n = 141)	P-value	Homeless (n = 118)	Non-homeless (n = 118)	P-value	Homeless (n = 23)	Non-homeless (n = 23)	P-value
Infectious	68 (48)	48 (34)	0.02	58 (49)	39 (33)	0.02	10 (43)	9 (39)	>0.9
Liver	23 (16)	33 (23)	0.18	16 (14)	30 (25)	0.03	7 (30)	3 (13)	0.3
Massive GI bleeding	8 (6)	5 (4)	0.57	7 (6)	2 (2)	0.2	1 (4)	3 (13)	0.6
Cardiovascular	19 (13)	25 (18)	0.41	18 (15)	23 (19)	0.5	1 (4)	2 (9)	>0.9
Oncology	7 (5)	17 (12)	0.053	7 (6)	14 (12)	0.2	0 (0)	3 (13)	0.23
Other	16 (11)	13 (10)	0.7	12 (10)	10 (8)	0.8	4 (17)	3 (13)	>0.9

In comparison to non-homeless patients, homeless patients were more likely to die because of infectious causes and less likely because of oncological problems (p = 0.02 and p = 0.05, respectively). There was no difference in liver, cardiovascular, and other mortality between homeless and non-homeless groups of patients. In the subgroup of men, homeless men frequently died because of infectious causes, and non-homeless men frequently died because of liver problems (p = 0.02 and p = 0.03, respectively). There was no difference in causes of mortality between homeless and non-homeless women.

Regarding specific causes of mortality, the most common cause of infectious mortality both in homeless and non-homeless men was pneumonia (60% and 51%, respectively), followed by urinary tract infection (16% and 18%, respectively) and foot gangrene (17% and 18%, respectively). There was no significant difference between specific causes of infectious mortality between both groups of patients (Table 3).

**TABLE 3:** Specific causes of infectious and liver mortality between homeless and non-homeless males (Bratislava, Slovakia. 2024).

Specific cause of death	Homeless patients	Non-homeless patients	P-value
Infectious	n = 58	n = 39	
Pneumonia, n (%)	35 (60)	20 (51)	0.4
Urinary tract infection, n (%)	9 (16)	7 (18)	0.8
Foot gangrene, n (%)	10 (17)	7 (18)	>0.9
Billiary tract infection, n (%)	1 (2)	0 (0)	>0.9
*Clostridium difficile* infection, n (%)	0 (0)	1 (3)	0.4
Spontaneous bacterial peritonitis, n (%)	0 (0)	2 (5)	0.2
Spondylodiscitis, n (%)	1 (2)	0 (0%)	>0.9
Necrotizing pancreatitis, n (%)	1 (2)	0 (0%)	>0.9
Infectious endocarditis, n (%)	1 (2)	2 (5%)	0.6
Liver	n = 16	n = 30	
Cirrhosis Child Pugh A, n (%)	0 (0)	0 (0)	>0.9
Cirrhosis Child Pugh B, n (%)	1 (6)	4 (13)	0.64
Cirrhosis Child Pugh C, n (%)	14 (87)	24 (80)	0.7
Alcoholic hepatitis, n (%)	6 (37)	5 (16)	0.2
MELD Na score, mean ± SD	26.5 ± 5.9	27.7 ± 8.5	0.6

In the liver subgroup, the most prevalent stage of cirrhosis in the homeless group on admission was Child-Pugh C, which does not differ significantly from the non-homeless group (87% vs. 80%, p = 0.69). The mean value of the MELD Na score in homeless men was 26.5 ± 5.9, which was not statistically different from the mean MELD NA score in non-homeless men (p = 0.57).

There was no difference between the number of patients with alcoholic hepatitis in both groups (37% vs. 16%, p = 0.15).

The autopsy examination was performed in 90 cases. In the cohort of homeless patients, the autopsy was conducted on 43 patients (30%), and in the cohort of non-homeless patients, on 47 patients (33%). According to the autopsy records, the three most common causes of death in homeless patients were infectious causes (45%), cardiovascular causes (17%) and liver causes (14%). In non-homeless patients, the three most common causes of death were infectious causes (28%), liver causes (26%), and cardiovascular causes (23%). There was no difference in age and sex in autopsied homeless and non-homeless patients. According to the Godman criteria, the absolute agreement between clinical and autopsy records (Class V) was found in 69% of cases, and major discrepancies (Class I and II) were found in 23%. There were no significant differences in selected Classes between homeless and non-homeless patients (see Table 4).

**TABLE 4:** Agreement between clinical and pathological diagnoses in homeless and non-homeless patients assessed by Goldman criteria (Bratislava, Slovakia. 2024).

Goldman criteria	Homeless (n = 47)	Non-homeless (n = 43)	P-value
Class I, n (%)	7 (15)	6 (14)	>0.9
Class II, n (%)	3 (6)	5 (12)	0.5
Class III, n (%)	2 (4)	1 (2)	>0.9
Class IV, n (%)	3 (6)	1 (2)	0.6
Class V, n (%)	32 (68)	30 (70)	>0.9
Class I + II, n (%)	10 (21)	11 (26)	0.8

## Discussion

In our study of homeless rough sleepers, the mean age at the time of death (56 years) was 20 years lower than in the non-homeless group of patients. The mean age at the time of death was approximately 8–10 years higher compared to other cohorts of homeless people from the United States [17], Canada [9], or Western Europe [18] and similar to cohorts of homeless people from Eastern Europe [19]. Men comprised the vast majority (83%) of the dead rough sleepers. The most common causes of death were infectious, liver, and cardiovascular problems. In the subgroup of men, homeless rough sleepers more frequently died because of infectious diseases in comparison to non-homeless sex- and age-matched controls, who more frequently died because of liver problems. However, the three most common causes of death (infectious, liver, cardiovascular) did not differ between groups. There was no significant difference in mortality between homeless and non-homeless women.

The majority of studies concerning the mortality of homeless people have been conducted in the United States and Western European states. In the United States, the major causes of death in a cohort of 1,302 homeless persons (between the years 2003-08) were drug overdose, cancer, and heart disease. In comparison to the years 1988-93, drug overdose replaced HIV as an emerging health problem [11]. In France, the major causes of death among homeless people between 2008-10 were external causes (e.g., assault, intentional self-harm, accidental drowning) and cancer. Notably, 28% of deaths have been assigned as “ill-defined and unknown” [18]. In a study from East London, England, the highest numbers of deaths were attributed to substance misuse, liver disease, and cardiac-related deaths [20]. It is important to note, however, that most of these studies were not limited to people admitted to the hospital for acute illness, and many homeless deaths (e.g., due to overdoses, homicide, suicide, and car crashes) do not occur in hospitals.

Little is known about the mortality of rough sleepers - a subgroup of homeless patients who primarily sleep outside. A study from Boston, Massachusetts, showed that the all-cause mortality rate for cohorts of unsheltered people was nearly three times higher than that of the rest of the homeless cohort. The most common causes of death in this cohort were cancer, alcohol use disorder, and chronic liver disease [21]. These patients have a unique blend of comorbidities and environmental factors, which predispose them to a higher risk of morbidity and mortality. In our cohort, more than 80% of rough sleepers abuse alcohol and cigarettes, and more than 10% use intravenous drugs. 16% of them had hypothermia, and 10% had ectoparasites - all factors associated with harsh environments and poor hygiene.

Outcomes of homeless and non-homeless acutely ill patients admitted to the hospital showed inconclusive results. Two studies, one from Korea [22] and one from France [23], showed that both homeless and non-homeless acutely ill patients have the same prognosis, and homelessness *per se* was not significantly associated with in-hospital mortality in multivariate analysis in these studies. However, living in the street was one of the factors independently associated with mortality in a study from France [23]. One small study from Canada with 63 sex- and age-matched acutely ill homeless patients showed that both cohorts had similar comorbidities except for increased alcohol and illicit drug use. Still, the cohort of homeless patients had significantly higher in-hospital mortality (29% vs. 8%) [9].

Regarding hospital mortality, inequality of management and care of homeless people could be a factor, especially with invasive procedures. Homeless adults hospitalized for acute myocardial infarction were less likely to undergo coronary angiography compared with non-homeless adults, percutaneous coronary intervention, and coronary artery bypass graft. What is more, the in-hospital mortality rate for most cardiovascular conditions was higher among homeless persons [10]. The stigma associated with homelessness could contribute to these disparities. For example, homeless people are often blamed for not adequately managing risks or safeguarding, above all, against communicable diseases, willfully putting themselves and others at risk of infection. Homeless people are, therefore, perceived as disaffiliated individuals who are irresponsible and deviant, representing disorder and requiring either elimination or containment [24]. In our cohort of rough sleepers, however, we did not find a significantly higher prevalence of communicable diseases like HIV, hepatitis B, hepatitis C, and syphilis in homeless people compared to matched non-homeless controls.

The stigma associated with homelessness did not play a significant role in our study. Patients in both groups have similar levels of care regarding intravenous vasopressors, antibiotics, degree of oxygen treatment, imaging, and invasive diagnostic procedures. What is more, the percentage of autopsies performed did not differ between groups, nor the agreement between clinical and pathological causes of death. However, the majority of homeless patients died in the first 5 days after hospital admission because of infectious diseases - implying that these patients came to the hospital in the late, developed stage of the disease. This is an important issue because infectious diseases belong to the so-called avoidable deaths - deaths that should not occur in the presence of timely and effective healthcare [25]. One of the causes of later presentation to the hospital could be the stigma associated with homeless people dealing with outpatient care [26]. Problems with the approachability of outpatient care could also explain the somewhat unexpected findings in our study, i.e., a lower number of comorbidities in roofless individuals. Similar results were, however, found in a study from Korea [22]. Upon admission, non-homeless patients were more likely to have diabetes, cirrhosis, or cancer compared to matched homeless patients [22]. The lower number of chronic comorbidities like diabetes, coronary artery disease, chronic pulmonary disease, or malignancy in homeless patients may be due to underdiagnosis rather than their “healthier” status compared to controls. A study from the United States showed that the homeless population had, in fact, a significantly higher cancer incidence rate compared to the general population, but in homeless patients, it was detected at a later stage in the course of the disease [27].

Another surprising finding could be no difference in intravenous drug users between homeless (11%) and non-homeless patients (6%). On the other hand, we found a significantly higher proportion of homeless patients who abuse alcohol (84% vs. 49%). According to the 2008 meta-analysis (5,684 unselected homeless individuals), the prevalence of intravenous drug users among homeless patients in our study was consistent with other countries (estimated prevalence ranged from 4.7% to 54.2%), and alcohol consumption far exceeded published results (estimated prevalence ranged from 8.1% to 58.5%) [28]. This finding could be connected with the increased affordability of alcohol in Slovakia, driven primarily by changes in the relative price of alcohol [29].

Our study has several limitations. The analyzed group of patients was small. We analyzed only homeless patients admitted to the one internal medicine department in one city—Bratislava. Thus, our results may not be generalizable for homeless patients in other Slovakian cities. Our study focused specifically on the causes of in-hospital mortality of people living in extreme forms of homelessness. Therefore, our results may not be generalizable for all homeless individuals. On the other hand, we analyzed a homogenous, clearly defined group of acutely ill roofless patients. In almost a third of the cohort, the clinical data were compared with autopsy records. Our study could add relevant clinical information to the field of clinical research, which has been rarely studied to date.

In conclusion, our study, for the first time, analyzed mortality patterns in homeless rough sleepers in Slovakia. This is also one of the few studies concerning homeless in-hospital mortality in Europe. Rough sleepers who died in the hospital are approximately 20 years younger than the mean age of death of hospitalized non-homeless patients. 83% of the rough sleepers are men, and 59% of the rough sleepers died before reaching old age (60+). The three most common causes of death did not differ between homeless and non-homeless matched cohorts. Homeless rough sleepers died most likely because of infectious diseases. Given the growing prevalence of homelessness in Slovakia as well as worldwide, evidence concerning this issue should be strengthened. Discussing data about the outcomes of homeless people could help to develop and implement better clinical as well as public health strategies, firstly, by helping to reduce the invisibility of the issue of massive premature mortality amongst homeless populations and roofless individuals, in particular.
